# *Eisenia bicyclis* Extract Ameliorates Colitis in In Vitro and In Vivo Models Through Modulation of mTOR Axis and Gut Microbiota Composition

**DOI:** 10.3390/foods14050714

**Published:** 2025-02-20

**Authors:** Qunzhe Wang, Yuri Im, Jumin Park, Hye Lim Lee, Dae Gon Ryu, Hyemee Kim

**Affiliations:** 1 Department of Food Science and Nutrition & Kimchi Research Institute, Pusan National University, Busan 46241, Republic of Korea; 1939104850jyb@gmail.com (Q.W.); yuripoku@naver.com (Y.I.); juminpark@pusan.ac.kr (J.P.); 2 BK21 FOUR Program: Precision Nutrition Program for Future Global Leaders, Pusan National University, Busan 46241, Republic of Korea; 3 Department of Internal Medicine, Medical Research Institute, Pusan National University School of Medicine and Research Institute for Convergence of Biomedical Science and Technology, Pusan National University Yangsan Hospital, Yangsan 50612, Republic of Korea; roasua@hanmail.net

**Keywords:** *Eisenia bicyclis*, ulcerative colitis, anti-inflammatory, mTOR axis, intestinal flora disturbance

## Abstract

Ulcerative colitis (UC) is a chronic inflammatory disease of the colon that is associated with dysbiosis in the gut microbiota. *Eisenia bicyclis*, a marine alga, is known for its anti-inflammatory, antioxidant, and gut microbiota-modulating properties. This study explored the mechanisms by which a 70% ethanol extract of *E. bicyclis* may alleviate UC, through both in vitro and in vivo experiments. LC-MS/MS analysis revealed eckol, 7-phloroeckol, dieckol, phlorofucofuroeckol A, and fucofuroeckol as key phenolic compounds present in the extract. The administration of *E. bicyclis* significantly improved symptoms in a dextran sulfate sodium (DSS)-induced colitis mouse model by reducing intestinal shortening, splenomegaly, and histological scores. Both cell and animal studies demonstrated that *E. bicyclis* suppressed the release of inflammatory cytokines, downregulated the mRNA expression of genes related to the mTOR pathway, and reduced the p-mTOR/mTOR ratio. Microbiota analysis revealed that, while the Firmicutes/Bacteroidetes ratio was elevated in UC mice, *E. bicyclis* administration normalized this imbalance, with a notable increase in the abundance of beneficial probiotics such as *Bifidobacterium bifidum*. In conclusion, a phenolic-rich extract of *E. bicyclis* demonstrates significant potential as a dietary supplement to prevent and mitigate UC by modulating both the mTOR signaling pathway and gut microbiota composition.

## 1. Introduction

Inflammatory bowel disease (IBD) is a persistent gastrointestinal disorder of unknown etiology, influenced by multiple factors. Ulcerative colitis (UC), a subtype of IBD, is characterized by inflammation confined to the colonic mucosa, leading to the formation of ulcers [1]. While UC is generally not life-threatening, it can result in severe complications, including significant bloody stools, colon perforation, severe dehydration, ocular inflammation, an increased risk of colon cancer, and toxic megacolon, as well as venous and arterial thrombosis [1]. In recent years, the incidence and prevalence of IBD have continued to rise in Asia, South America, and Southern and Eastern Europe [2]. The principal medications currently used to treat UC all have severe adverse effects [3], highlighting the need for alternative therapeutic approaches. The identification of natural foods or supplements with proven efficacy and minimal side effects is therefore of critical importance.

Seaweed, particularly brown algae, contains a high concentration of phenolic compounds such as phlorotannins, fucoxanthin, and polysaccharides like fucoidan. These compounds exhibit high antioxidant capacity, as well as anti-inflammatory, anticancer, and immune-modulating properties [4]. *Eisenia bicyclis* is a brown alga harvested primarily in cold waters off the coast of East Asia and utilized in Korean and Japanese cuisines. *E. bicyclis* has been reported to exhibit various biological activities, including antioxidant, antimicrobial, anti-inflammatory, anti-diabetic, and gut-modulating properties [5,6,7,8,9]. It is rich in phlorotannins, such as phloroglucinol (PG), fuhalols, phlorethols, dieckol, and eckol, as well as other compounds like laminarin and fucoidan [7,10]. Eckol and dieckol, two unique components of brown algae, have also been shown to inhibit UC in animal experiments [11]. Studies have shown that eckol can increase the abundance of probiotics such as *Lactobacillus* and *Bifidobacterium* in an experimental colitis model, and has physiological activities such as regulating the intestinal flora, antioxidant effects, and anti-inflammatory effects [12]. Dieckol inhibits oxidative stress and the progression of DSS-induced experimental colitis by inhibiting the NF-κB inflammatory pathway and inducing the Nrf2/HO-1 signaling pathway [13].

mTOR is a serine–threonine protein kinase regulated by various cellular signals, and most upstream input signals are controlled through PI3K/Akt signal transduction [14]. The PI3K/Akt/mTOR pathway is involved in multiple cellular functions like cell growth, proliferation, autophagy, apoptosis, and tumor cell migration, and plays a key role in various diseases and cancers. The activation of the PI3K/Akt/mTOR pathway leads to increased drug resistance in tumor cells, inhibits tumor cell apoptosis, and promotes tumor cell survival [14]. There are data showing that inhibiting the PI3K/Akt/mTOR pathway can inhibit the development of various diseases, including ischemic brain injury, neurodegenerative diseases, leukemia, diabetes, etc. [15,16]. The mTOR axis can stimulate IKK activation, thereby activating NF-κB [17]. The transcription factor NF-κB is regarded as a key regulator of IBD and UC, and its activation leads to the release of a large number of inflammatory factors and an increase in intestinal tissue inflammation [18]. Therefore, inhibiting the activation of the PI3K/Akt/mTOR pathway and the activation of NF-κB may be key for reducing colon inflammation in UC and promoting intestinal cell survival. Studies have shown that some phenolics may suppress the occurrence and progression of UC by inhibiting the mTOR axis; however, studies involving *E. bicyclis* or eckol and dieckol with the mTOR axis have not been conducted.

The integrity of the intestinal mucosal epithelial barrier is crucial in the onset and progression of IBD. As IBD progresses, the extent of damage to the intestinal mucosal barrier increases, and this damage, in turn, exacerbates the disease [19]. The integrity of the intestinal mucosal barrier is also dependent on its interaction with the gut microbiota [20]. Therefore, the gut microbiota is considered a key environmental factor in the etiology of UC [21]. In patients with IBD, the gut microbiota is often characterized by a high microbial load and reduced diversity, with significant reductions in beneficial bacteria such as Firmicutes and Bacteroidetes, and an increase in pathogenic bacteria like *Enterobacteriaceae* [21]. *Clostridium coccoides* and *Clostridium leptum* clusters were found to be significantly low in fecal samples of patients with UC, as were the levels of short-chain fatty acids (SCFAs), particularly n-butyrate and isobutyrate [22]. Recent studies have shown that polyphenols, such as catechins, anthocyanins, and ellagic acid, along with other phenolic compounds, can promote the growth of beneficial lactic acid bacteria, including *Faecalibacterium* and *Bifidobacterium* [22]. Phlorotannins, which are unique phenolic compounds found in brown algae, have also been extensively studied. Phlorotannins increase the synthesis of SCFAs, protect against the invasion of pathogens, and maintain the integrity of the intestinal mucosal barrier and normal intestinal function [23]. Therefore, the brown alga *E. bicyclis* was chosen in this study to investigate its prebiotic effect on the gut microbiota.

Our research laboratory previously conducted a study (Go et al. (2024)), which focused on extracting tannins from *E. bicyclis* and comparing the effects of tannins alone versus tannins fermented with *Lactobacillus* on colitis [24]. However, further experiments were deemed necessary to investigate the impact of *E. bicyclis* on gut microbiota composition and its role in modulating inflammatory responses. Therefore, in this study, we aimed to explore the effects of *E. bicyclis* ethanol extract on anti-inflammatory activity, intestinal barrier protection, and gut microbial dynamics. *E. bicyclis* was hypothesized to protect the integrity of the intestinal barrier and reduce inflammation by inhibiting mTOR axis-related genes, inhibiting the phosphorylation of NF-κB, and regulating the intestinal flora to prevent experimental UC.

## 2. Materials and Methods

### 2.1. Preparation of E. bicyclis Extract

*E. bicyclis* (EB) in its raw form was sourced from Ulleungdo Mall (http://www.ulleungdomall.com/). The extraction method was as follows: The washed *E. bicyclis* was air-dried and then completely dried using a freeze dryer. The dried material was ground into a powder, soaked in 10-fold g/volume of 70% ethanol for 24 h, a process that was repeated three times, and the extract was collected by filtration. The extract was evaporated at 36 °C and completely dehydrated using a FDU-2100 freeze dryer (RIKAKIKAI Co., Ltd., Tokyo, Japan) at −80 °C for 3 days [25]. To ensure the biological activity of the extract, the dried material was ground into a fine powder using a mechanical grinder after freeze-drying and stored at −22 °C in a sealed container to maintain stability and prevent oxidation. The EB exhibited a yield of 25.3 g/100 g. Additionally, a stability test was conducted using the Folin–Ciocalteu assay throughout the experimental period, confirming that the total phenolic content remained stable [26]. The total flavonoid content of the extract was measured using diethylene glycol colorimetry [27].

### 2.2. Liquid Chromatography–Tandem Mass Spectrometry Conditions

The main phenolic substances in the extract were measured using LC-TOF-MS/MS analysis, performed on an Agilent integrated system (Agilent Technologies, Santa Clara, CA, USA) coupled to a hybrid quadrupole time-of-flight (Q-TOF) mass spectrometer. Chromatographic separation was performed using a Waters-BEH C18 column (2.1 × 100 mm, 1.8 μm particle size; Waters, Milford, MA, USA). Elution was performed at 40 °C with a flow rate of 0.4 mL/min, and spectral data were collected at 330 nm. Regarding the gradient elution conditions, 0.1% formic acid (FA) in water and acetonitrile were used for gradient elution as follows: 0–2 min, 98% in water and 2% in acetonitrile; 2–20 min, gradually changing to 100% acetonitrile solution; 20–25 min, 0% water solution and 100% acetonitrile solution; 25-25 min, gradually change to 98% water solution and 2% acetonitrile solution; 26–30 min, 98% water solution and 2% acetonitrile solution. Mass spectrometric analysis was conducted using the ZenoTOF™ 7600 system (SCIEX, Framingham, MA, USA) in both positive- and negative-ion mode. The TOF MS survey scan range was configured from 60 to 1250 Da, while the MS/MS-dependent scan range covered 50 to 1200 Da. Phlorotannins, polymers composed of multiple phloroglucinol units linked by carbon–carbon bonds, may exhibit fragmentation patterns in mass spectrometry similar to those of phloroglucinol. Therefore, phlorotannins are usually analyzed using phloroglucinol as a standard [28]. A standard solution of phloroglucinol was injected into the LC-MS/MS system, and the peak areas were plotted to generate a calibration curve for phloroglucinol. The peak areas of each analyte in the sample were then measured and converted to concentrations using the calibration curve. The concentration of each component C_x_, calculated as phloroglucinol equivalents, is determined by the following equation:

$$C_{x}=\frac{\;\mathrm{Peak}\;\mathrm{area}\;\mathrm{of}\;x}{\mathrm{Peak}\;\mathrm{area}\;\mathrm{of}\;\mathrm{phloroglucinol}}\times \frac{\mathrm{Molecular}\;\mathrm{weight}\;\mathrm{of}\;\mathrm{phloroglucinol}}{\mathrm{Molecular}\;\mathrm{weight}\;\mathrm{of}\;x}$$

### 2.3. Cell Culture

RAW264.7 and Caco-2 cells were supplied by the Korean Cell Line Bank (Seoul, Korea). RAW264.7 cells were cultured in RPMI-1640 (HyClone, Logan, YT, USA) supplemented with 10% (*v*/*v*) fetal bovine serum (FBS, HyClone) and 1% (*v*/*v*) penicillin (HyClone) in a humidified environment containing 5% CO_2_ at 37 °C. Caco-2 cells were cultured in MEM (HyClone) containing 20% (*v*/*v*) FBS, 1% (*v*/*v*) penicillin, 1% (*v*/*v*) sodium pyruvate, and 1% (*v*/*v*) 50× MEM amino acid solution. The EB extract concentrations of 25 mg/L and 50 mg/L were prepared by weighing the freeze-dried EB extract powder. The measured amounts were then dissolved in DMSO to ensure a homogeneous solution and subsequently mixed with PBS to create a final solution containing 25% DMSO in PBS. Cell viability was determined via an MTT assay using 3-(4,5-dimethylthiazol-2-yl)-2,5-diphenyltetrazolium bromide [29]. Caco-2 and RAW264.7 cells were plated in 12-well plates and cultured for 24 h. After removing the culture medium, 0.5 mL of EB solution (25 and 50 mg/L, prepared in 25% DMSO in PBS) was added to each well and incubated for 1 h. Lipopolysaccharide (LPS; 50 ng/mL for RAW264.7 cells, 2 µg/mL for Caco-2 cells, Escherichia coli O55:B5, Sigma-Aldrich, St. Louis, MO, USA) was used to induce inflammation in the positive control group, while 25% DMSO in PBS was used as the negative control [30]. Following this, 0.5 mL of a second solution containing EB (at the same concentrations) and LPS was added to the wells and incubated for 3 h to facilitate RNA extraction. For protein extraction, Caco-2 cells were seeded in 6-well plates at a density of 5 × 10^4^ cells/mL per well. The treatment protocol remained consistent, except that the EB and LPS-containing solution was incubated for 20 h.

### 2.4. Animal Experimental Design

Male BALB/c mice (6–7 weeks old) were obtained from OrientBio (Seongnam-si, Gyeonggi-do, Korea) and housed for a week. To induce colitis, dextran sodium sulfate (DSS; MP Biomedicals, Solon, OH, USA) was administered, as DSS-induced mouse colitis models are widely used in IBD research, particularly for studying UC. In this study, a 2.5% DSS administration for 7 days was used, following previous protocols such as that of Chassaing et al. (2014). This model effectively induces acute colitis, mimicking clinical symptoms observed in human UC, including inflammation, mucosal damage, bleeding, weight loss, and colon shortening [31]. Therefore, this model is widely used in research on gut microbiota, immune responses, and related mechanisms [32,33]. After stabilization for one week, the mice were separated into four groups by body weight (bw), with eight mice per group: control, DSS, DSS-EB-L (100 mg/kg/day), and DSS-EB-H (200 mg/kg/day). Mice were orally administered EB as the experimental group or PBS as the negative control group every day for four weeks. On the 22nd day of the experiment, 2.5% DSS was added to their drinking water to induce colitis. Weight change, vitality, blood in the stool, defecation, and anal bleeding were monitored and recorded to determine the disease activity index (DAI) score. At the end of the experiment, all mice were sacrificed, and blood, cecum, colon, and spleen were harvested for subsequent analyses [34].

### 2.5. Histopathological Testing

The segment of the large intestine distal to the mouse near the anus was paraffin-embedded, stained with hematoxylin and eosin (H&E), and observed under a BX50 fluorescence microscope (Olympus, Tokyo, Japan). The histological score was obtained by adding the inflammation score and the ulcer score. The inflammation score is defined as follows: 0—complete epithelial tissue without inflammatory cell infiltration; 1—mild epithelial hyperplasia and mucosal and submucosal inflammatory cell infiltration; 2—epithelial hyperplasia and inflammatory cell infiltration in the mucosa and submucosa; 3—mucosal and submucosa pseudopolyps and severe inflammatory cell infiltration. Ulcer score was defined as follows: 0—no ulcer and intact mucosal tissue; 1—superficial epithelial inflammation but normal mucosal crypts; 2—partial loss of mucosal crypts and erosion of epithelial tissue; 3—complete disappearance of mucosal crypts and ulceration of epithelial tissue [35].

### 2.6. RT-PCR

Total RNA was extracted from cells and tissues using the RNeasy Mini Kit (QIAGEN, Hilden, Germany), with Trizol supplementation for colon extraction. RNA concentration was measured using a spectrophotometer and then balanced. cDNA reverse transcription was performed using the iScript cDNA Synthesis Kit (Bio-Rad, Hercules, CA, USA), followed by DNA amplification on the CFX Connect Real-Time PCR System (Bio-Rad, Hercules, CA, USA). Primer sequences are provided in Supplementary Table S1. β-Actin was used as a reference. All reagents used are referred to in previous studies [36].

### 2.7. ELISA Assay

The ELISA method used in the cell experiment was described in a previous study [37]. In the in vivo test, serum was used to measure lipocalin 2, while colon tissue was homogenized in lysis buffer (T-PER™, Thermo Fisher, Waltham, MA, USA), and then centrifuged at 10,000× *g* for 5 min at 4 °C to obtain the supernatant for inflammatory factor analysis, including IL-6, TNF-α, IFN-γ, MPO, and IL-17. The ELISA kits were obtained from R&D Systems (Minneapolis, MN, USA).

### 2.8. Western Blot for Protein Detection

After protein concentration was adjusted, samples were separated by sodium dodecyl sulfate–polyacrylamide gel electrophoresis (SDS-PAGE), and then transferred to polyvinylidene difluoride (PVDF) membranes using the Trans-Blot Cell program (30 V) running for 1 h (Bio-Rad). After blocking, the membranes were incubated with primary antibodies targeting mTOR, p-mTOR, NF-κB, p-NF-κB, S6K, p-S6K, and β-actin (Cell Signaling Technology, Danvers, MA, USA) for 16 h at 4 °C. Unbound primary antibodies were then washed followed by incubation with secondary antibodies for 1 h at room temperature. The intensity of the Western blot was measured using a chemiluminescent imaging system (Davinch K, Seoul, Republic of Korea).

### 2.9. Gut Microbiota Analysis

The gut microbiome composition was analyzed as described in a previous study [36]. In brief, total DNA was extracted from fecal samples using the PowerFecal Pro DNA Kit (QIAGEN, Hilden, Germany), and DNA concentration was determined with a Qubit 4 fluorometer (Thermo Fisher, Waltham, MA, USA). For 16S rRNA sequencing, microbial DNA from two mice per group was pooled in equal proportions (n = 3–4/group) and subjected to sequencing on the Illumina iSeq platform (San Diego, CA, USA). The V4 region of the 16S rRNA gene was amplified using the following primers: forward primer (TCGTCGGCAGCGTCAGATGTGTATAAGAGACAGCCTACGGGNGGCWGCAG) and reverse primer (GTCTCGTGGGCTCGGAGATGTGTATAAGAGACAGGACTACHVGGGTATCTAATCC). Raw sequencing reads were processed for quality control and adapter trimming using Trimmomatic (v0.39), followed by taxonomic classification and diversity analysis using QIIME2 (v1.9.5) (https://qiime2.org, accessed on 5 July 2022). For bacterial species identification, qPCR (CFX System; Bio-Rad) was performed with selected bacterial primers, which are listed in Supplemental Table S2 (Macrogen; Seoul, Korea). The relative abundance of these species was calculated as a proportion of total bacterial content using the F341/R518 primer pair [38].

### 2.10. Statistical Analysis

Data analysis was conducted using GraphPad Prism 8 software (GraphPad Software, La Jolla, CA, USA), and results are presented as mean ± SD. One-way ANOVA with Dunnett’s post hoc test was used to calculate p-values, while the abundance of specific bacteria was assessed using the nonparametric Mann–Whitney U test. Statistical significance was defined as *p* < 0.05.

## 3. Results

### 3.1. Phenolic Composition and Content in E. bicyclis Extract

**Figure 1:**
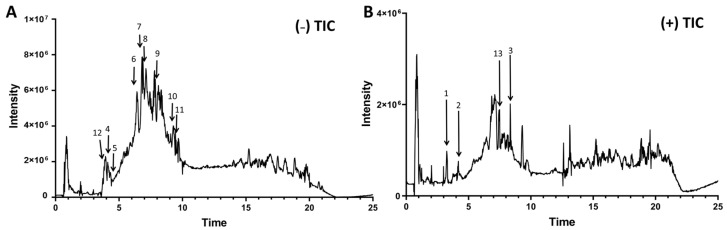
Representative LC-MS/MS chromatograms of *E.bicyclis* extract. (**A**) Negative-ion TIC and (**B**) positive-ion TIC of the EB extract: 1. phloroglucinol; 2. fucodiphlorethol G; 3. dioxinodehydroeckol; 4. diphlorethol; 5. bifuhalol; 6. eckol; 7. 7-phloroeckol; 8. 2-O-(2,4,6-Trihydroxyphenyl)-6,6′-bieckol; 9. dieckol; 10. phlorofucofuroeckol A; 11. fucofuroeckol; 12. 3,4-dihydroxybenzoic acid; and 13. zingerol. EB: *E. bicyclis* extract.

Using the Folin–Ciocalteu assay and diethylene glycol colorimetry, the phenolic content in EB was determined to be 407.63 ± 6.01 mg of phloroglucinol equivalent (PGE)/g, and the flavonoid content was 323.24 ± 6.06 mg of catechin equivalent (CE)/g. The LC-MS/MS results of EB are presented in Figure 1 and Table 1. Notably, the primary phenolic components identified in EB include eckol, 7-phloroeckol, dieckol, phlorofucofuroeckol A, and fucofuroeckol. These results align with previous studies reporting that eckol, dieckol, and phlorofucofuroeckol A are the predominant phlorotannins in *E. bicyclis*, with similar concentration ranges [24]. For example, Lim at al. (2024) reported dieckol levels to be 16.5 mg/g biomass [39], which is comparable to our study, where the dieckol concentration was 16.53 µg PGE/mg. The ethanol extraction yield was also higher than that in the previous paper. Under the negative-ion total-ion chromatogram, the majority of components are more easily identified.

### 3.2. Effects of E. bicyclis Extracts in LPS-Stimulated Cell Models

**Figure 2:**
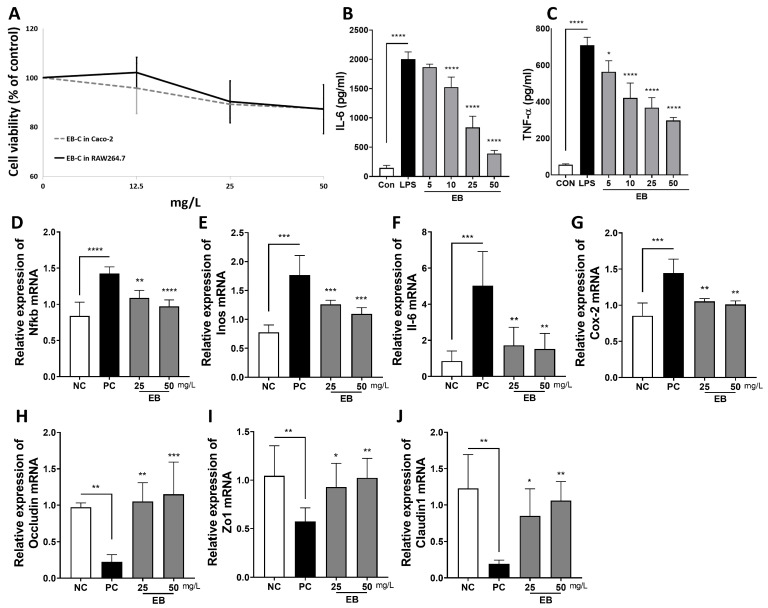
Inflammation-reducing effects of *E. bicyclis* extract (EB) in LPS-stimulated Caco-2 and RAW264.7 cells. (**A**) Cytotoxicity of EB (0–50 mg/L) against Caco-2 and RAW264.7 cells was measured using MTS assay. (**B**) IL-6 and (**C**) TNF-α levels in RAW264.7 cells treated with LPS (50 ng/mL) and EB. (**D**) Nfkb, (**E**) Inos, (**F**) Il6, and (**G**) Cox-2 mRNA expressions in RAW264.7 cells treated with LPS (50 ng/mL) and EB. (**H**) Occludin, (**I**) Zo1, and (**J**) claudin-1 mRNA expressions in Caco-2 cells treated with LPS (2 μg/mL) and EB. The data are shown as mean ± SD (n = 4), with comparisons made to the positive control (PC) group. Statistical significance was defined as * *p* < 0.05, ** *p* < 0.01, *** *p* < 0.001, and **** *p* < 0.0001. NC: negative control; PC: positive control; EB: *Ecklonia bicyclis* extract.

After 48 h of treatment with 25 and 50 mg/L EB, the cell viability of RAW264.7 cells was 90.28% and 87.25%, respectively, while that of Caco-2 cells was 89.18% and 81.97% (Figure 2A). In LPS-treated RAW264.7 cells, IL-6 levels decreased by 23.81%, 58.16%, and 80.51%, while TNF-α levels were reduced by 40.62%, 48.05%, and 58.02% with 10, 25, and 50 mg/L EB, respectively (Figure 2B,C). In the cellular inflammation model, the mRNA expression of Nfkb, Inos, Cox-2, and IL-6 was significantly increased in RAW264.7 cells stimulated by LPS, whereas these expressions were significantly downregulated following EB treatment (Figure 2D–G). Tight junctions, which regulate intestinal permeability, are one of the key targets in ulcerative colitis. In the LPS-stimulated Caco-2 inflammation model, LPS treatment led to a reduction in the mRNA expression of occludin, Zo-1, and claudin-1 in Caco-2 cells, whereas EB treatment increased their mRNA levels (Figure 2H–J).

### 3.3. Effects of E. bicyclis Extract on mTOR Axis in LPS-Treated Caco-2 Cells

**Figure 3:**
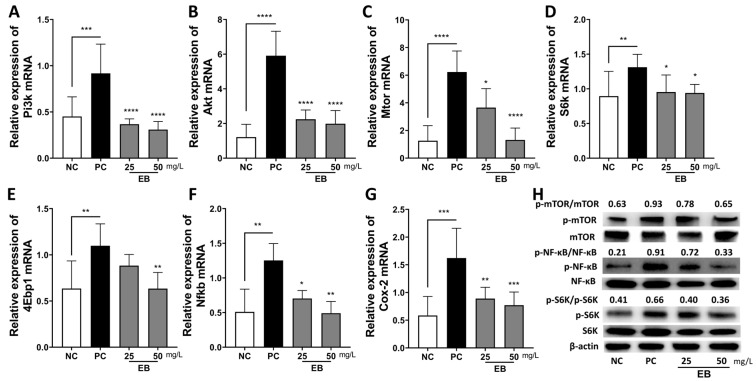
*E. bicyclis* extract (EB) alters cellular responses in LPS-induced Caco-2 cells. (**A**) Pi3k, (**B**) Akt, (**C**) Mtor, (**D**) S6k, (**E**) 4Ebp1, (**F**) Nfkb, and (**G**) Cox2 mRNA expressions in LPS (2 μg/mL)-stimulated Caco-2 cells with EB extracts (25 and 50 mg/L) for 4 h. β-Actin was used as the reference gene. The data are shown as mean ± SD (n = 4), with comparisons made to the positive control (PC) group. Statistical significance was defined as * *p* < 0.05, ** *p* < 0.01, *** *p* < 0.001, and **** *p* < 0.0001. (**H**) Protein expressions of p-mTOR, mTOR, p-NF-κB, NF-κB, p-S6K, S6K, and β-actin in LPS (2 μg/mL)-treated Caco-2 cells with EB extracts (25 and 50 mg/L) for 24 h. NC: negative control; PC: positive control; EB: *Ecklonia bicyclis* extract.

To elucidate the mechanism by which EB regulate inflammation, the expression of genes involved in inflammation and the mTOR axis was measured in LPS-treated Caco-2 cells. The mRNA expression of Pi3k, Akt, Mtor, S6k, 4epb1, and the inflammation-associated genes Nfkb and Cox-2 significantly increased after LPS treatment, whereas they significantly decreased after EB treatment. Moreover, the inhibitory effect was discovered to be more pronounced in the high-concentration EB treatment (Figure 3A–G). In addition, the protein levels of p-mTOR, p-S6K, and p-NF-κB in Caco-2 cells were significantly increased after LPS stimulation; however, the levels of these proteins were notably reduced when EB was treated (Figure 3H).

### 3.4. Effects of E. bicyclis Extract on Histological Changes in DSS-Treated Mice

**Figure 4:**
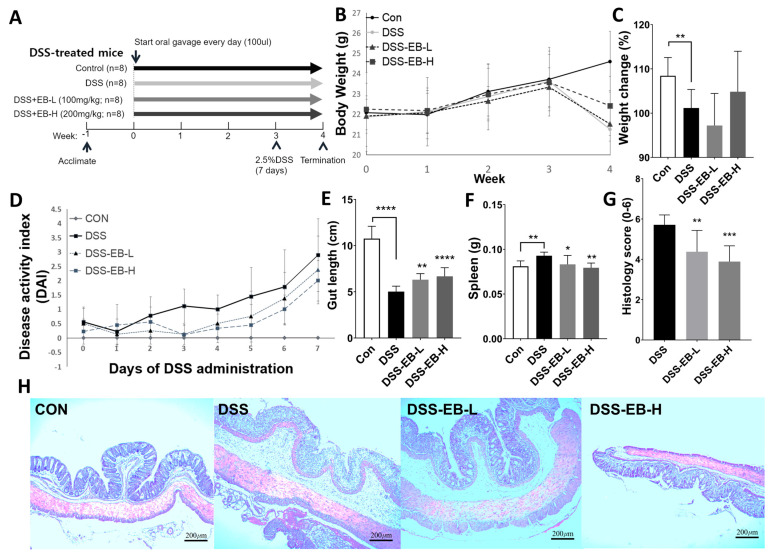
Effects of *E. bicyclis* extract (EB) on sign of inflammation in UC mice. (**A**) Experimental timeline of the DSS-treated mice. (**B**) The weight changes of mice were recorded weekly. (**C**) Percentage change in body weight. (**D**) Changes in DAI scores per group after administration of DSS. (**E**) Gut length and (**F**) spleen weight were compared between the four groups. (**G**) Histological score of colon tissues stained with hematoxylin and eosin (H&E) was assessed by scoring the level of inflammation and ulceration on a scale of 0–6. (**H**) Representative images of colon tissues (magnification ×400). Data are represented as mean ± SD (n = 8/group). Significance is denoted by * *p* < 0.05, ** *p* < 0.01, *** *p* < 0.001, and **** *p* < 0.0001 (in comparison to the DSS-treated group).

Throughout the animal experiment, overall physiological status, biochemical markers, and organ histopathology evaluations indicated that the EB groups demonstrated better health outcomes than the DSS group, with no observed side effects from extract administration, such as weight loss or diarrhea. In the DSS-induced colitis in mice, the DSS group had 8% lower body weight than the control group, whereas the EB-treated group did not show significant results (Figure 4B,C). The DAI also showed no significant changes after EB administration (Figure 4D). However, intestinal lengths that were shortened and spleens that were enlarged by DSS treatment in comparison with control mice were significantly improved in the EB-treated group (Figure 4E,F). According to the histological scores, EB administration significantly reduced intestinal inflammation and ulceration induced by DSS (Figure 4G). H&E staining of the colons of mice in the DSS group revealed severe tissue damage, as evidenced by severe colonic ulceration, inflammatory cell infiltration, crypt damage, and nearly total loss of tissue structure. In the EB-treated groups, inflammatory cell infiltration and partial tissue damage were significantly reduced compared with those in the DSS group (Figure 4H).

### 3.5. Effects of E. bicyclis Extract on Inflammatory Biomarkers in DSS-Exposed Mice

**Figure 5:**
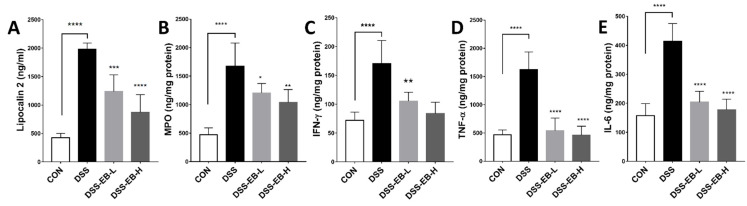
Effects of *E. bicyclis* extract (EB) on inflammatory markers in UC mice. (**A**) Lipocalin-2 levels in serum and (**B**–**E**) myeloperoxidase (MPO), IFN-γ, TNF-α, and IL-6 levels in colon tissue were quantified using ELISA analysis. Data are represented as mean ± SD (n = 8/group). Significance is denoted by * *p* < 0.05, ** *p* < 0.01, *** *p* < 0.001, and **** *p* < 0.0001 (relative to the DSS-treated group).

Lipocalin 2, also known as neutrophil gelatinase-associated lipocalin (NGAL), is considered a biomarker of diseases, including inflammation, tumors, infection, and kidney injury, is detectable in urine, blood, and stool [40], and has been proposed as a biomarker of active UC [41]. Myeloperoxidase (MPO), an essential iron-containing lysosomal enzyme present in myeloid cells, is secreted by macrophages. Previous studies have indicated its use as a biomarker for the diagnosis of IBD, as it is overexpressed in many inflammatory diseases [42]. Inflammatory cytokines, including IL-6, IL-1β, TNF-α, and IFN-γ, are also included among the markers of UC [43]. In this experiment, serum lipocalin-2 levels and the concentrations of MPO, TNF-α, IFN-γ, and IL-6 in colon tissue were significantly higher in the DSS group, with a notable reduction observed following EB treatment (Figure 5E).

### 3.6. E. bicyclis Extract Improves Colitis in Mice by Inhibiting the mTOR Axis

**Figure 6:**
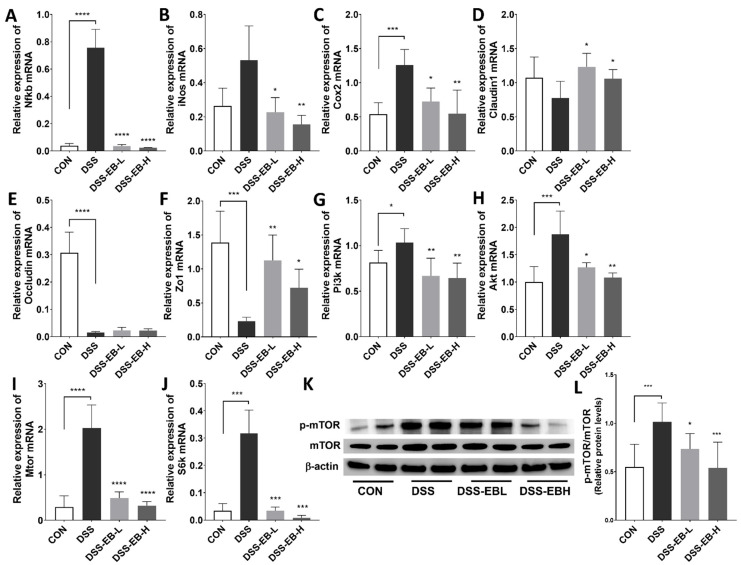
*E. bicyclis* extract (EB) effects on tight junctions and mTOR pathway markers in UC mice. (**A**–**J**) Nfkb, Inos, Cox2, claudin-1, occludin, Zo1, Pi3k, Akt, Mtor, and S6k mRNA levels in colon tissue. β-Actin was used as the reference gene. (**K**) p-mTOR and total mTOR protein expression in mice colon tissue. (**L**) p-mTOR/mTOR ratio was calculated. Data are represented as mean ± SD (n = 8/group). Significance is denoted by * *p* < 0.05, ** *p* < 0.01, *** *p* < 0.001, and **** *p* < 0.0001 (in comparison to the DSS-treated group).

DSS exposure increased the mRNA expression of inflammatory markers Nfkb, Inos, Cox-2, and the PI3K/Akt/mTOR pathway in the colon tissue of mice, whereas EB administration significantly inhibited this phenomenon in a concentration-dependent manner (Figure 6A–C,G–J). The expression of tight junction-related genes, such as ZO-1 and claudin-1, was significantly suppressed in the DSS group, whereas it was significantly enhanced in the EB groups (Figure 6D–F). The expression of p-mTOR in the DSS group was significantly increased and was significantly decreased after EB administration. The calculation of p-mTOR/mTOR ratio revealed this situation more clearly, and the high-dose group had a greater inhibitory effect (Figure 6K,L).

### 3.7. Effects of E. bicyclis Extract on Gut Microbiota

**Figure 7:**
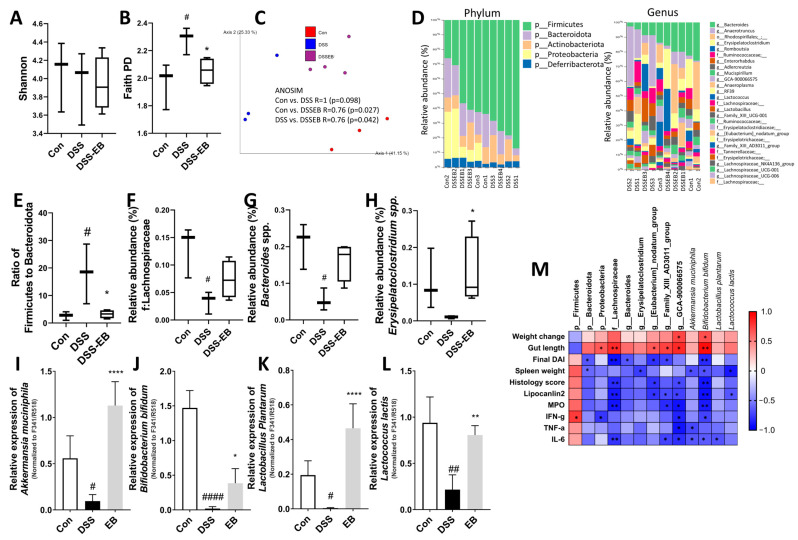
Impact of *E. bicyclis* extract (EB) on the composition and distribution of gut flora in UC mice. (**A**) Shannon index and (**B**) Faith PD for evaluating alpha-diversity. (**C**) Principal coordinate analysis (PCoA) of unweighted UniFrac distances. (**D**) Taxonomic analysis of microbiota at the phylum and genus levels. (**E**) The relative abundance of *Firmicutes* to *Bacteroidota.* (**F**–**H**) Abundance differences of specific microbial groups between DSS and EB groups. Data are expressed as box and whisker plots (n = 3 or 4; fecal DNA from 2 mice was pooled into one sample for 16S sequencing). (**I**) Quantitative PCR results for *Akkermansia muciniphila*, (**J**) *Bifidobacterium bifidum*, (**K**) *Lactobacillus plantarum*, and (**L**) *Lactococcus lactis*. The relative abundance of bacterial groups was represented as the ratio of total bacteria (F341/R518). Data are expressed as mean ± SD (n = 8/group). Compared between the control and the DSS group, # *p* < 0.05, ## *p* < 0.01, and #### *p* < 0.0001. Compared between the DSS group and the EB group, * *p* < 0.05, ** *p* < 0.01, and **** *p* < 0.0001 by nonparametric Mann–Whitney U test. (**M**) Spearman correlation analysis was performed to assess the relationship between gut microbiota species and UC-related indices (* *p* < 0.05, ** *p* < 0.01).

In the 16s sequencing results, no significant differences in Shannon index were observed between the DSS-administered and EB-administered groups, and the Faith PD α-diversity was high in the DSS group, which lowered after EB intake (Figure 7A,B). β-Diversity analysis revealed statistically significant differences in bacterial distribution between groups (ANOSIM: Con vs. DSS R = 1 [*p* = 0.098], Con vs. DSS-EB R = 0.76 [*p* = 0.027], and DSS vs. DSS-EB R = 0.76 [*p* = 0.042]; Figure 7C). In the taxonomic analysis of bacteria at the phylum and genus levels (Figure 7D), the ratio of Firmicutes and Bacteroidetes was significantly high following DSS treatment, which reduced after EB administration (Figure 7E). In addition, DSS treatment decreased the abundance of the Lachnospiraceae family and *Bacteroides* spp., whereas EB treatment showed an increasing trend (Figure 7F,G). *Erysipelatoclostridium* spp., a Gram-positive bacterium, is a commensal bacterium in the normal human gut that regulates dyslipidemia and has anti-aging effects [44,45]. The abundance of *Erysipelatoclostridium* spp. decreased after DSS treatment and significantly increased after EB ingestion (Figure 7H). After screening the abundance of well-known probiotics at the species level using qPCR, the abundance of *Bifidobacterium bifidum*, *Lactobacillus plantarum*, *Lactococcus lactis*, and *Akkermansia muciniphila* significantly decreased in UC mice and increased after administration of a high concentration of EB (Figure 7I–L). When calculating the correlation between 16S sequencing results and inflammation-related indices, the *Lachnospiraceae* family level, Family_XIII_AD3011_group, and *B. bifidum* exhibited negative correlations with several inflammation-related indicators (Figure 7M).

## 4. Discussion

This study identified the chemical components of *E. bicyclis* extract extracted using ethanol and found that it was rich in eckol, 7-phloroeckol, dieckol, phlorofucofuroeckol A, and fucofuroeckol. Previous studies [24] showed that ethanol may be better for extracting *E. bicyclis*’ phenolic substances. Cell and mouse experiments have shown that *E. bicyclis* extract can prevent experimental ulcerative colitis by inhibiting the PI3K/Akt/mTOR pathway, inhibiting inflammation, and regulating the intestinal microbial composition. The hypothesis that *E. bicyclis* extract can reduce the intestinal inflammatory response and regulate the intestinal microbiota through the synergistic effects of various phenolic and flavonoid compounds has been confirmed.

For inflammatory bowel disease (IBD) research, the most commonly used in vitro models include RAW264.7 macrophages and intestinal epithelial cells such as Caco-2, HT-29, and T84 cells [46]. Among these, Caco-2 cells are widely utilized because they closely mimic intestinal cells in their characteristics, including tight junctions, apical brush borders, and the expression of enterocyte-specific enzymes and transporters [47]. Therefore, in this study, RAW264.7 macrophages and Caco-2 cells were selected to establish in vitro inflammatory models through LPS induction, allowing for the evaluation of the inhibitory effects of EB on the intestinal inflammatory response and its protective role in the mucosal barrier. Polyphenols are known to exert anti-inflammatory effects by inhibiting mTOR activity, thereby reducing the expression of inflammatory markers [48]. In a related study on a DSS-induced ulcerative colitis (UC) model, inhibition of the mTOR axis significantly reduced the expression of TNF-α, IL-1β, and iNOS, leading to improved conditions [49]. These findings are consistent with the results of this study, which demonstrated that EB exhibited strong anti-inflammatory activity and inhibitory effects on the PI3K/Akt/mTOR pathway in the Caco-2 cell inflammation model by measuring cellular RNA and protein levels. Tight junctions between intestinal epithelial cells are critical for maintaining the integrity of the intestinal mucosal barrier, preventing endotoxins from entering the bloodstream, and ensuring proper nutrient transport [50]. In the Caco-2 cell inflammation model, EB treatment significantly restored tight junction proteins such as occludin and claudin-1. Claudin-1 is crucial for maintaining barrier function, while occludin contributes to tight junction structure and regulates paracellular barrier function. The expression and structural integrity of these proteins, along with ZO-1, directly impact gut barrier functionality [51,52]. Disruption of the intestinal barrier increases permeability, a characteristic of many inflammatory diseases [53]. Additionally, in the RAW264.7 macrophage inflammation model, EB treatment reduced the RNA expression of pro-inflammatory mediators (including iNOS, NF-κB, and IL-6) and the protein levels of TNF-α and IL-6. These results indicate that EB inhibits the inflammatory response and protects the intestinal mucosal barrier by suppressing mTOR activation, enhancing tight junction protein expression, and downregulating NF-κB and related inflammatory factors.

The chemically induced DSS model is one of the most frequently used animal models for colitis [31]. Its histopathological changes resemble those of UC, including the ulceration and erosion of colonic tissue, the disappearance of crypts, inflammation of the colonic mucosa with massive granulocyte infiltration, elevated myeloperoxidase, weight loss, and shortened colon length [54,55]. In this study, DSS-treated mice exhibited significant body weight loss, spleen enlargement, intestinal shortening, ulceration, erosion of colonic tissue, neutrophil infiltration, and loss of crypts. Pathological manifestations of colitis were significantly reduced in the EB groups. Moreover, compared with the DSS group mice, the EB-administered group showed significantly reduced levels of pro-inflammatory factors. Lipocalin-2, a potent bacteriostatic protein, is regulated by Th17 cell immune responses and functions as an inflammatory marker [56]. MPO is a marker of neutrophil infiltration [57]. EB administration significantly reduced the amount of lipocalin-2 secreted by Th1 cells, MPO secreted by neutrophils, and pro-inflammatory cytokines TNF-α, IFN-γ, and IL-6 released by macrophages [58]. This suggests that EB can improve inflammation in DSS-induced colitis by inhibiting the infiltration of neutrophils, Th1 cell activity, and the production of pro-inflammatory mediators. In addition, the expression of inflammation- and mTOR axis-related mRNAs and proteins was significantly higher in the DSS group than in the control group, which was reduced following EB administration. S6K is the downstream target of mTOR. The PI3K/AKT/mTOR pathway is a key signaling cascade that is overactivated in inflammatory diseases and cancers, leading to exacerbated immune responses and chronic inflammation. PI3K phosphorylates membrane phospholipids to activate AKT, which inhibits the suppressor protein Rheb, ultimately activating mTOR. mTOR then phosphorylates downstream effectors such as S6K1 and 4E-BP1, regulating cell growth, protein synthesis, and immune responses [59,60]. *E. bicyclis* extract, particularly its phlorotannin compounds (e.g., eckol, dieckol), has been reported to inhibit the phosphorylation of PI3K and AKT, thereby suppressing mTOR activation [61,62]. Additionally, mTOR interacts with the NF-κB pathway, which amplifies inflammatory signaling [63]. Since *E. bicyclis* extract has been shown to inhibit NF-κB translocation and reduce the expression of pro-inflammatory cytokines (e.g., TNF-α, IL-6) [64], this suggests a secondary mechanism through which mTOR activity is suppressed. Furthermore, the modulation of gut microbiota by *E. bicyclis*—notably increasing the abundance of beneficial bacteria such as *Lactobacillus, Bifidobacterium*, and *A. muciniphila*—may enhance the production of SCFAs, which have been reported to inhibit mTOR signaling via AMPK activation and GPCR-mediated pathways [65]. These findings indicate that *E. bicyclis* modulates the mTOR axis through direct inhibition of PI3K/AKT signaling, suppression of NF-κB activation, and gut microbiota-driven SCFA-mediated inhibition of mTOR. However, further research is needed to fully elucidate the molecular mechanisms underlying these interactions. Based on the results from cell experiments, this animal study focused on mTOR analysis via Western blotting, which aligned with the cell experiment findings. It was confirmed that EB inhibits mTOR activity, leading to a reduction in NF-κB pathway activity, as well as a reduction in the secretion of iNOS, COX-2, and inflammatory cytokines. Although this study did not include a direct comparison with conventional colitis treatments, the findings suggest that EB extract may act through mechanisms similar to well-established therapies, such as mesalazine (5-ASA), corticosteroids, and biological agents (e.g., TNF-α inhibitors like infliximab and adalimumab), which have been shown to modulate the mTOR pathway in colitis models [66]. While these drugs effectively reduce inflammation, their long-term use is often associated with significant side effects. In contrast, EB extract presents a novel therapeutic approach by targeting multiple inflammatory pathways while potentially offering a more favorable safety profile. The active compounds in EB, particularly phlorotannins such as eckol and dieckol, may exert anti-inflammatory effects through PI3K/AKT/mTOR inhibition, NF-κB modulation, and gut microbiota regulation, highlighting its potential as an alternative or complementary treatment for colitis. EB administration also improved the expression of tight junction proteins in DSS-induced colonic injury, maintaining the integrity of the intestinal mucosal barrier against exposure to external substances, including inflammatory mediators and potential toxins. Although Western blot analysis for tight junction proteins was not performed in the animal study, the combination of in vivo and in vitro RT-PCR results, along with TEER measurements of intestinal tissue permeability in the co-culture model, provides sufficient evidence to suggest that EB helps to maintain the integrity of the intestinal barrier [67]. These results suggest that the administration of EB could prevent UC by inhibiting the PI3K/Akt/mTOR pathway, preserving the expression of tight junction proteins, intestinal function, and the intestinal barrier. 

The homeostasis of the intestinal flora is closely linked to host health. Although it is uncertain whether intestinal disturbances cause or are a consequence of IBD, there is a clear connection between gut microbiota dysfunction, dysbiosis, and IBD [21]. In patients with IBD, stool microbiota analysis revealed that the biodiversity and composition differed from those of healthy individuals, with lower abundance of *Eubacterium* and *Akkermansia*, which produce butyrate and propionate [68,69]. Polymeric polyphenols can reportedly escape the human body’s digestion and enter the intestinal tract, where they interact with the gut microbiota, acting as prebiotics and boosting the production of SCFAs [70]. In addition to suppressing inflammation and modulating the gut microbiota, some research indicates that phlorotannins also positively protect the gut barrier [71]. Therefore, EB is believed to have a positive effect on IBD prevention by modulating the gut microbiota composition. This experiment revealed that EB did not increase the abundance and uniformity of bacteria; however, it altered their distribution significantly. The results indicate that EB administration significantly reduced the ratio of *Firmicutes* to *Bacteroidetes*, which was increased by DSS. *Firmicutes* is positively correlated with IFN-γ, and Bacteroidetes is negatively correlated with the final DAI score and spleen weight; given that Firmicutes are often associated with less beneficial metabolic profiles [72], EB is suggested to ameliorate UC. EB treatment also restored the abundance of Lachnospiraceae, *Bacteroides* spp., *Erysipelatoclostridium* spp., *A. muciniphila*, *B. bifidum*, *L. plantarum*, and *L. lactis*, which were reduced by DSS and are negatively correlated with the secretion of inflammatory mediators and pathological damage in UC. Probiotics such as Lachnospiraceae, *B. bifidum*, *A. muciniphila*, *L. plantarum*, and *L. lactis* have been extensively studied and have been shown to increase SCFAs, improve the intestinal mucosal barrier, degrade mucin, inhibit the NF-κB pathway, have anti-obesity effects, inhibit the progression of atherosclerosis, and decrease inflammatory mediators [70]. Among them, *L. plantarum* and *B. bifidum* have been shown to prevent chronic diseases such as type 2 diabetes, obesity, non-alcoholic fatty liver, kidney stones, and cardiovascular diseases in clinical trials [73]. Another *L. lactis*, which is also commercially available, has the ability to prevent and treat inflammatory and autoimmune diseases in addition to the above-mentioned abilities [74]. Therefore, EB is speculated to increase the production of small-molecule organic acids, such as SCFAs, by regulating the intestinal flora, maintaining the health of intestinal cells, and inhibiting inflammation to prevent UC.

In in vitro experiments, anti-inflammatory effects were observed at 25 and 50 mg/L. However, due to the complex physiological differences between the in vitro and in vivo systems, a further literature review was conducted to determine the appropriate dosage for animal experiments. We referred to previous studies that used doses ranging from 50 to 200 mg/kg body weight (bw), which demonstrated significant effects on colitis-related parameters, such as reduced inflammation and increased intestinal length, at doses within this range [71,75]. Based on these findings, we selected 100 and 200 mg/kg as the experimental doses. However, because our results did not show a statistically significant difference between the doses, it is possible that the selected concentrations were relatively high or that a plateau effect occurred at these concentrations. According to our LC analysis, the intake levels of eckol in our study were 2–4 mg PGE/kg bw, while the dieckol intake was 1.7–3.4 mg PGE/kg bw. Previous studies have reported that eckol at 0.5–1 mg/kg bw and dieckol at 5–15 mg/kg bw were effective in colitis models [12,13]. This suggests that, while our eckol concentration may be relatively high, the dieckol concentration is within the effective range reported in previous studies. Based on these observations, future studies will perform dose–response assessments with fractionated tannins to refine the optimal concentration range and evaluate their specific biological effects. Notably, the 200 mg/kg dose, which led to changes in gut microbiota distribution, corresponds to a human dosage of approximately 16.2 mg/kg when converted based on body surface area [76]. This equates to a daily dosage of 1134 mg for an adult male weighing 70 kg and includes 22.8 mg PGE of eckol and 18.75 mg PGE of dieckol per day.

The timing of DSS administration presents a limitation. DSS was provided during the final week of the experiment, after which the study was terminated, and no significant differences in body weight or DAI were observed. It is believed that different results might have been obtained if the DSS treatment had been followed by a recovery period. In addition, this study lacks mechanistic validation regarding the role of EB in regulating mTOR signaling and gut microbiota modulation in colitis improvement. To further confirm the role of EB, additional experiments are necessary using antibiotic-fed mice and mice administered an mTOR inhibitor to assess the inhibitory effect of EB on colitis while minimizing the influence of mTOR signaling and gut microbiota regulation. This would help determine whether the anti-inflammatory effects of EB extracts are primarily mediated through gut microbiota or if they occur independently of mTOR signaling.

## 5. Conclusions

The phenolic extract of *E. bicyclis* contains key bioactive compounds, including eckol, 7-phloroeckol, dieckol, phlorofucofuroeckol A, and fucofuroeckol, and has demonstrated anti-inflammatory effects in both in vitro and in vivo colitis models. The extract effectively attenuated intestinal mucosal damage and suppressed inflammatory mediator release by inhibiting the mTOR/NF-κB pathway. Additionally, it increased the abundance of beneficial gut microbiota, such as Lachnospiraceae, *B. bifidum, L. plantarum*, and *A. muciniphila*, which are known to have protective effects against ulcerative colitis. Given its anti-inflammatory and prebiotic properties, *E. bicyclis* extract shows potential as a functional food ingredient or prebiotic for the prevention and management of ulcerative colitis, particularly for patients with chronic inflammatory bowel disease. However, the study lacked mechanistic validation of mTOR signaling and gut microbiota modulation, necessitating further studies using mTOR-inhibitor and antibiotic-treated mouse models to clarify the extract’s anti-inflammatory mechanism. Additionally, the potential toxicity of *E. bicyclis* extract at high doses remains unclear, requiring further toxicological assessments to establish its optimal and safe dosage range for clinical applications.

## Figures and Tables

**Table 1:** Comprehensive chemical characterization of *E. bicyclis* extract using LC-MS/MS analysis.

				EB Extract		
Component Name		Ion Type	Formula	RT (min)	μg PGE/mg *	*m*/*z*
Phlorotannins						
1	Phloroglucinol	[M+H]+	C_6_H_6_O_3_	3.2	8.22	127.03
2	Fucodiphlorethol G	[M+H]+	C_24_H_18_O_12_	4.16	0.77	499.08
3	Dioxinodehydroeckol	[M+H]+	C_18_H_10_O_9_	8.49	0.60	371.03
4	Diphlorethol	[M−H]−	C_12_H_10_O_6_	4.11	0.12	249.04
5	Bifuhalol	[M−H]−	C_12_H_10_O_7_	4.62	0.43	265.03
6	Eckol	[M−H]−	C_18_H_12_O_9_	6.81	20.16	371.04
7	7-Phloroeckol	[M−H]−	C_24_H_16_O_12_	6.89	18.55	495.05
8	2-O-(2,4,6-Trihydroxyphenyl)-6,6′-bieckol	[M−H]−	C_42_H_26_O_21_	7.17	1.42	865.09
9	Dieckol	[M−H]−	C_36_H_22_O_18_	8.32	16.53	741.07
10	Phlorofucofuroeckol A	[M−H]−	C_30_H_18_O_14_	9.31	15.13	601.06
11	Fucofuroeckol	[M−H]−	C_24_H_14_O_11_	9.71	9.71	477.04
**Etc.**						
12	3,4-Dihydroxybenzoic acid	[M−H]−	C_7_H_6_O_4_	3.95	0.25	153.01
13	Zingerol	[M+H]+	C_11_H_16_O_3_	7.44	3.42	197.11

* μg phloroglucinol equivalent (PGE)/mg extract.

## Data Availability

The original contributions presented in this study are included in the article/Supplementary Material. Further inquiries can be directed to the corresponding authors.

## Supplementary Materials

The following supporting information can be downloaded at https://www.mdpi.com/article/10.3390/foods14050714/s1, Table S1: Sequences of quantitative RT-PCR primers; Table S2: Sequences of primers used for bacterial profiling. Refs. [77,78,79,80,81] are cited in Supplementary Materials.
