# Supplementary material for: Eisenia bicyclis Extract Ameliorates Colitis in In Vitro and In Vivo Models Through Modulation of mTOR Axis and Gut Microbiota Composition
Source: Foods. 2025 Feb 20;14(5):714. doi: 10.3390/foods14050714 (PMC11899094; doi:10.3390/foods14050714)
Supplement: Supplementary file 1 [file foods-14-00714-s001.zip › foods-3402518-supplementary.pdf]

Supplemental Table S1. Sequences of quantitative RT-PCR primers.

Gene	Forward primer	Reverse primer
<u>*Homo sapiens*</u>		
β-actin	GGACTTCGAGCAAGAGATGG	AGCACTGTGTTGGCGTACAG
COX2	TGCTGTGGAGCTGTATCCTG	GAAAACCCACTTCTCCACCA
NOS2 (iNOS)	ACAAGCCTACCCCTCCAGAT	TCCCGTCAGTTGGTAGGTTC
RELA (NFkB p65)	GCGAGAGGAGCACAGATACC	CTGATAGCCTGCTCCAGGTC
PIK3R1 (PI3K)	TCACTACCGCCACGAGTCTCTG	ACTGCCTCCACGCTGTCCTC
AKT1	AGCACCACAGCCACCCAGTC	TCATCCTCGTCCTCCTCGTTGTC
MTOR	CTTGCTGAACTGGAGGCTGATGG	CCGTTTTCTTATGGGCTGGCTCTC
S6K	GCACAGCAAATCCTCAGACA	TTGGAGATCATGGGAAAAGC
ZO-1	AACTGGGCTCTTGGCTTGCTATTC	TCCAGAAGTCAGCACGGTCTCC
Claudin-1	GCTGTGGATGTCCTGCGTGTC	GAGGATGCCAACCACCATCAAGG
Occludin	AACTTCGCCTGTGGATGACTTCAG	GACCTTCCTGCTCTTCCCTTTGC
<u>*Mus musculus*</u>		
β-actin		
Cox2	AGAAGGAAATGGCTGCAGAA	GCTCGGCTTCCAGTATTGAG
Nos2	TTCCAAGAGCCTTGATGTTT	GTAGGTAAGGGCGTTGGTCA
Rela (NFkb p65)	GCGTACACATTCTGGGGAGT	ACCGAAGCAGGAGCTATCAA
Il6	AGTTGCCTTCTTGGGACTGA	CAGAATTGCCATTGCACAAC
Pik3r1 (PI3K)	GCGTGACATGTAGGCTCTCA	CAGTTTCCTTGGCTTTGCTC
Akt1	CACACAGCTGGAGAACCTCA	AGGGAACACACAGGAAGTGG
Mtor	CGCTACTGTGTCTTGGCATC	GGTTCATGCTGCTTAGTCGG
S6k	CAGAACAGGGGGTGCATAGT	CTGAGGAAGGAAGCCACTTG
Zo-1	GCTAAGAGCACAGCAATGGA	GCATGTTCAACGTTATCCAT
Occludin	CCCAGGTGGCAGGTAGATTA	GCACCACGTTGGAAAAGAAT
Claudin-1	TTTTCCCGATGACCTTTCTG	AGTTTGCAGGATCTGGGATG

Supplemental Table S2. Sequences of primers used for bacterial profiling.

**Target**	**Forward primer**	**Reverse primer**	**Reference**
Uni (F341/R518)	CCTACGGGAGGCAGCAGT	ATTACCGCGGCTGCTGG	Lubbs (2009)
Akkermansia muciniphila	CTGAACCAGCCAAGTAGCG	CCGCAAACTTTCACAACTGACTTA	Collado et al. (2007)
Bifidobacterium bifidum	ATTTGAGCCACTGTCTGGTG	CATCCGGGAACGTCGGGAAA	Sul (2007)
Lactobacillus plantarum	CCGTTTATGCGGAACACCTA	TCAGGATTACCAAACATCAC	Bartkiene (2016)
Lactococcus lactis	TGAAGAATTGATGGAACTCG	CATTGTGGTTCACCGTTC	Achilleos (2013)
